# Applicability and effectiveness of ultrasound combined with nerve stimulator-guided lumbosacral plexus block in the supine versus lateral position during surgeries for lower limb fracture-a prospective randomized controlled trial

**DOI:** 10.1186/s12871-022-01710-9

**Published:** 2022-06-03

**Authors:** Yuting Xu, Jie Song, Xiaoqiong Xia, Xianwen Hu, Yawen Li, Yongbo Yu, Liang Wang, Zhiguo Tao

**Affiliations:** 1 Department of Anesthesiology, Chaohu Hospital of Anhui Medical University, Chaohu, Anhui China; 2 Department of Anesthesiology, The Second Hospital of Anhui Medical University, Hefei, Anhui China

**Keywords:** Nerve block, Nerve stimulation, Ultrasound, Lumbosacral plexus block, Lower extremity

## Abstract

**Background:**

Patients with lower limb fracture often have acute pain and discomfort from changes in position, and such pain affects early postoperative recovery. This study aimed to compare the applicability and effectiveness of ultrasound combined with nerve stimulator-guided lumbosacral plexus block (LSPB) in the supine versus lateral position during lower limb fracture surgery.

**Methods:**

We included 126 patients who underwent elective internal fixation for lower limb fracture who were divided into the S group and the L group by the random number table method and underwent LSPB guided by ultrasound combined with a nerve stimulator in the supine and lateral positions, respectively. The primary outcome was the dose of sufentanil used in surgery. The secondary outcomes were the maximum VAS (visual analogue scale) pain score at position placing for LSPB, the time of position placing, the time for nerve block,the number of puncture attempts,the haemodynamic indicators, the VAS score at 1, 12, and 24 h following surgery, postoperative satisfactory degree to analgesia and adverse events related to nerve block.

**Results:**

There was no statistically significant difference in dose of sufentanil used between the two groups(*P* = 0.142). The maximum VAS pain score at position placing(*P* < 0.01), the time of position placement(*P* < 0.01), the time for lumbar plexus block and the time of puncture attempts were significantly lower in the S group than in the L group (*P* < 0.01). However, the time for sacral plexus block was higher in the S group than in the L group (*P = 0.029*). There was no significant difference in haemodynamic indicators,number of puncture attempts for the sacral plexus, postoperative VAS scores, postoperative satisfactory degree to analgesia or adverse events related to nerve block between the two groups (all *P* > 0.05).

**Conclusions:**

Our study provides a more comfortable and better accepted anaesthetic regimen for patients undergoing lower limb fracture surgery. LSPB in the supine position is simple to apply and has definite anaesthetic effects. Additionally, it has a high level of postoperative analgesia and therefore should be widely applied.

**Trial registration:**

The trial was registered prior to patient enrolment at the Chinese Clinical Trail Registry (Date:11/03/2021 Number: ChiCTR2100044117).

**Supplementary Information:**

The online version contains supplementary material available at 10.1186/s12871-022-01710-9.

## Introduction

Lower limb fractures account for approximately one third of all fractures and may result in substantial mortality and morbidity [1]. The most common anatomic position of lower limb fracture is the ankle joint, which accounts for 22.6% of all lower limb fractures, followed by the tibia/fibula (17.3%), hip joint (16.7%) and tarsal/metatarsal bone (16.7%). Fractures of the hip, femur and other parts account for approximately 25% of all fractures [2].

Rapid urbanization and accelerated ageing of the population in China have led to a rapid increase in the number of patients with lower limb fractures caused by traffic injuries, architecture injuries and senile osteoporosis. The increase in residual injuries and disability increases the potential life loss and thus has become an important public health concern [3].

Patients with lower limb fractures have severe pain, and another 30% have moderate pain [4]. Inadequate pain control can lead to an altered release of hormones including insulin and catecholamines, metabolic disturbances, increased myocardial oxygen demand, agitation, delirium, delayed wound healing, hypoxia/atelectasis, and neuropsychiatric complications such as isolation, anxiety, and PTSD, which can lead to chronic pain [5]. Acute preoperative pain affects the duration of hospitalization and early mobilization and increases the risk of respiratory and cardiac complications [4]. Therefore,reducing patients’ preoperative pain and effectively managing perioperative pain are essential.

Over the recent years, lumbosacral plexus block (LSPB) has been widely applied in orthopaedics departments due to its advantages, including reduction in the application of opiates, decreasing the occurrence of acute pain, promoting early activation and shortening the time of hospital stay [6]. LSPB is a peripheral regional technique of anaesthesia and analgesia, that provides a block of the main components of the lumbosacral plexus. Fascia iliaca compartment block (FICB) is an anterior approach to lumbar plexus block [7]. FICB combined with sacral plexus block, can satisfy the needs of surgical anaesthesia below the hip. Ultrasound-guided nerve blocks with nerve stimulators increase the success rate and reduce risks, such as nerve injuries, undesirable spread-haematoma and renal puncture [8]. Conventional LSPB is mainly performed in the lateral position, while only very few studies have reported LSPB in the supine position. Therefore, the present study aimed to compare the impact of supine and lateral LSPB on primary outcome:sufentanil consumption, secondary outcomes including pain score,nerve block indicators, haemodynamic indicators, postoperative satisfactory degree to analgesia and adverse events related to nerve block in patients undergoing surgeries for lower limb fractures.

## Methods

This randomized, blinded study was performed in compliance with the Declaration of Helsinki and its amendments and was conducted according to the principles of Good Clinical Practice. The trial was registered prior to patient enrolment at the Chinese Clinical Trail Registry (11/03/2021 ChiCTR2100044117). The present study was approved by the Ethics Committee of the Chaohu Hospital Affiliated to Anhui Medical University (01/12/2020 202,001-KYXM-01) and all patients signed the relevant informed consent form. A total of 126 patients with lower limb fractures were recruited at the Department of Anesthesiology in two affiliated hospitals of Anhui Medical University (Chaohu Hospital, Chaohu; and the Second Hospital, Hefei) between March 2021 and June 2021.

### Randomization and blinding

We collected 66 cases in Chaohu Hospital and 60 cases in the Second Hospital, and in both hospitals we took the following approach for randomization. An independent anaesthetist,who was not involved in data management and statistical analyses numbered patients from 1 to 66/60 and then generated random numbers (in a 1:1 ratio) with a block size of 4 using the website www. Randomization. com. Then,all selected random numbers were sequenced from lowest to highest. Those with numbers 1 ~ 33/1 ~ 30 were considered the S group, and those with numbers 34 ~ 66/31 ~ 60 were considered the L group. The results of randomization were sealed in patient numbered envelopes and stored by the primary investigator until the end of the study or clinical emergency. The patients, the investigators responsible for postoperative follow up and the statisticians were all blinded to the randomization until the final statistical analyses were completed.

### Inclusion and exclusion criteria

The inclusion criteria used were as follows: 1) patients diagnosed with unilateral femoral neck fracture or lower fractures by X-ray or CT examination; 2) patients who consented to participate in the study and signed the relevant informed consent form); 3) patients with available complete clinical data; 4) patients with the capability of communication, expression and comprehension; 5) aged 18–75 years old; and 6) American Society of Anesthesiologists’ (ASA) physical status I-II.

The exclusion criteria were as follows: 1) patients with mental disorders or psychonosema; 2) puncture site infection; 3) patients with coagulation disorders; 4) patients who refused to participate or withdrew due to personal reasons; and 5) allergy to local anaesthetics.

### Treatment methods

#### Methods of anaesthesia

After entering the anaesthesia preparation room, patients were monitored with electrocardiography (ECG),heart rate (HR),blood pressure (BP), and pulse oxygen saturation (SpO_2_) and venous access was established. Prior to establishing venous access, an intravenous infusion of dexmedetomidine (Yangzijiang Pharmaceutical Group Co. Ltd.; SFDA approval number: H20183220) was performed at a rate of 300 μg/h for 10 min to induce full sedation of the patients. Subsequently, LSPB was performed under the guidance of an ultrasound combined nerve stimulatorusing a nerve stimulator (Stimuplex HNS 12, B. BRAUN, Germany) with a 5–10 MHz high-frequency linear array probe and a 2–5 MHz low-frequency convex array probe (FUJIFILM SonoSite, Bothell, WA98021 USA). Ropivacaine was obtained from Xianju Pharmaceutical Group Co. Ltd. (Zhejiang; SFDA approval number: H20163208).

#### Treatment of the S group

**Fig. 1:**
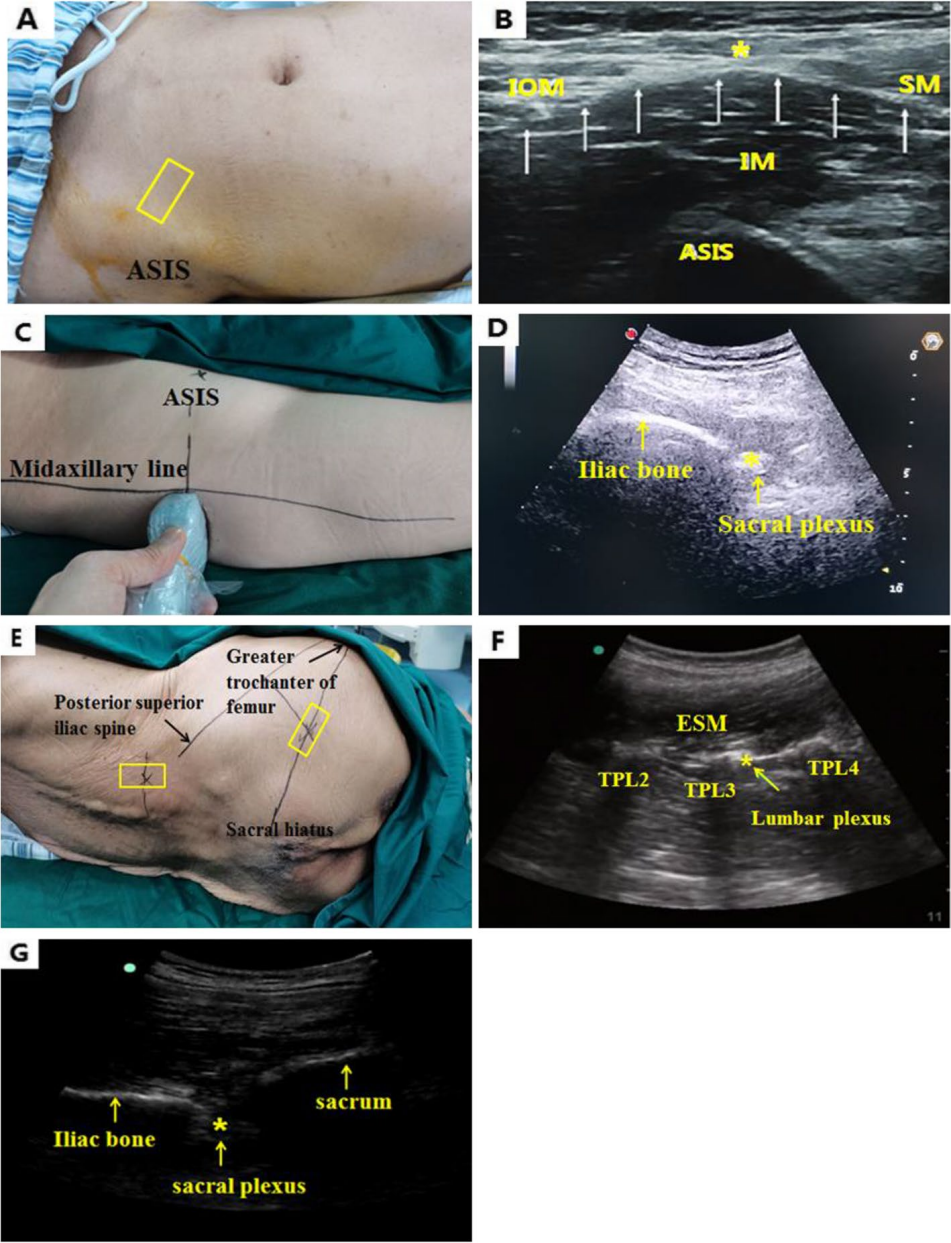
**A** A patient in the S group who was in the supine position for iliac plexus block; **B** ultrasound image of the patient (**A**) who underwent iliac plexus block. **C** A patient in the S group who was in the supine position for sacral plexus block; **D** ultrasound image of the patient (**C**) who underwent sacral plexus block. **E** A patient in the L group in the lateral position for LSPB; **F** ultrasound image of the patient (**E**) who underwent iliac plexus block. **G** Ultrasound image of the patient in the L group who underwent sacral plexus block. ASIS: anterior superior iliac spine; IOM: internal oblique muscle; SM: sartorius muscle; IM: iliacus muscle; ESM: erector spinae muscle; TP: transverse process; ultrasound probe

The anterior approach block of the lumbar plexus and supra-inguinal fascia iliaca compartment block were performed for patients in the S group. In brief [9], the superior anterior spine was touched and the high-frequency linear array probe was placed on the sagittal plane to acquire images of the anterior superior spine (Fig. 1A). The probe was slid inward to detect the iliac muscle. Subsequently, the probe was adjusted for ultrasound anatomy, including the detection of subcutaneous tissues, obliquus internus abdominis, sartorius muscle, iliac fascia, and iliac muscle (Fig. 1B). Intraplane insertion of the needle was performed by inserting a 21-G 100-mm needle to puncture the iliac fascia. The needlepoint was allowed to reach the site below the iliac fascia and no gas or blood was found in the back-pumping. A total of 5 ml normal saline was injected to clarify the site of the needle point. After the site was considered appropriate, the water separation technique was adopted by gradual injection of 30 ml 0.3% ropivacaine into the superficial and deep sites of the iliac muscle to expand the iliac fascia. For sacral plexus block in the supine position [10], the patients adopted the supine position with the affected side uplifted by 15°. A line from the anterior superior spine was made vertical to the midaxillary line and the intersection point was considered the positioning point (Fig. 1C). The maximal axis of the low-frequency convex array probe was vertical to the midaxillary line, after which the probe was slid from the head end to the tail end and was terminated when the iliac bone continuously appeared. The sacral plexus nerve was in the hyperecho area posterior to the iliac bone (Fig. 1D). Extra-plane needle insertion was performed. Under the guidance of ultrasound, the needlepoint was inserted towards the sacral plexus, whereas the initial current of the nerve stimulator, which was set at 1 mA, could induce the movement of tensor fasciae latae when reaching the sacral plexus. Subsequently, the current was adjusted to 0.5 mA and the needle was slowly inserted. When contraction of calf muscles was induced and back-pumping indicated a lack of gas or blood, 20 ml 0.3% ropivacaine was injected [11, 12].

#### Treatment of the L group

The lumbar plexus block for patients in the L group was performed according to previous studies [13]. The patients were placed in the lateral position and the skin and ultrasound probe were disinfected. The maximal axis of the low-frequency convex array probe was placed at the L3–4 space and at 4 cm parallel to the posterior midline of the spine (Fig. 1E). The ultrasound image indicated a trident-shaped structure formed by the transverse processes of three lumbar vertebrae. The transverse processes of L3–4 were positioned and the ultrasound probe was moved until the midpoint of the line between L3 and L4 transverse processes was located on the midline of the screen of the ultrasound apparatus (Fig. 1F). Extra-plane needle insertion was performed at the midpoint of the ultrasound probe until the needle point reached 1.5 cm below the transverse process and until it was superior to the musculiinter transversarii laterals. The nerve stimulator was connected to the nerve for nerve stimulation. Following contraction of the quadriceps femoris muscle, the current was adjusted to 0.5 mA; in cases of absence of blood or cerebrospinal fluid (indicated by back-pumping) and lack of contraction of the quadriceps femoris muscle, 30 ml ropivacaine (0.3%) was injected and intermittent back-pumping was performed to avoid intravenous injection. The midpoint of the line from the upper margin of the greater trochanter of the femur was marked to the posterior superior iliac spine to induce the sacral plexus block in a lateral position [14], from which a vertical line was made (an inner-downwards line). This line met the line from the greater trochanter of the femur to the sacral hiatus, and the intersection was the positioning point for the sacral plexus (Fig. 1E). The probe was routinely disinfected and subsequently placed at this intersection with the low-frequency convex array probe parallel to the line between the greater trochanter of the femur and the sacral hiatus. The image of the slope-shaped sacroiliac joint was displayed by ultrasound and the probe was slid along the line towards the tail end until the sacroiliac joint disappeared. Subsequently, sonographic images of the ischium on the outer side and of the sacrum on the inner side were displayed, and the hyperecho between the ischium and sacrum was the sacral plexus (Fig. 1G). The sacral plexus block was performed under the assistance of a nerve stimulator as conducted in the patients of the S group.

All nerve blocks were performed by the same experienced attending anaesthetis. Following completion of the nerve block in both groups, all patients received standardized general anaesthesia as follows: induction with propofol 2 mg/kg, sufentanil 0.2 μg/kg, and cis-atracurium 0.15 mg/kg. Intubation was performed via laryngeal mask based on a bispectral index (BIS) value of < 60 to allow autonomous or controlled respiration. Then, anaesthesia was maintained with propofol 6 mg/(kg·h), and the infusion rate of propofol was adjusted to keep the BIS within 40–60. The respiratory parameters were adjusted to maintain 35–45 mmHg of P_ET_CO_2_ (partial pressure of end-tidal carbon dioxide). According to the haemodynamics, 5 μg sufentanil was added if the heart rate or arterial pressure was increased by 15% in the surgery. Following surgery, patient-controlled intravenous analgesia (PCIA) (8–10 mg butorphanol + 10 mgazasetron, diluted to 100 ml) was used for analgesia, with aninitial dose of 2 ml, background dose of 2 ml/h, PCA dose of 2 ml and limiting dose of 18 ml/h.

The operation was performed by four medical groups in the two hospitals.

### Evaluation indicators

An investigator recorded (1) the maximum VAS pain score at position placing for LSPB, time of position placing, time for nerve block and a number of puncture attempts during the nerve block; (2) subsequently the dose of sufentanil used and haemodynamic indicators during the surgery were recorded; (3) the VAS score at 1, 12, and 24 h following surgery, postoperative satisfactory degree to analgesia, nerve block results and adverse events were also recorded in the two groups.

The following evaluation indicators were used. The primary outcome was the dose of sufentanil used in surgery. The secondary outcomes were as follows: the maximum VAS pain score at the position placed for LSPB (1–10 points: 0 points indicated no pain and 10 points indicated drastic pain); the time of position placement, the time for nerve block (from skin anaesthesia, ultrasound imaging, to the completion of local anaesthetic injection) the number of puncture attempts (each withdrawal of the needle to adjust the direction was considered as one attempt of puncture); the haemodynamic indicators, including heart rate, arterial pressure and the observation time including the time of entering the operating room (T0), completion of a nerve block (T1), skin incision (T2), skin suturing (T3) and 30 min following completion of the surgery (T4); the postoperative VAS score at 1, 12, and 24 h following surgery, with higher scores indicating more severe pain;the postoperative satisfactory degree to analgesia, where the scores ranged from 1 to 4 points (1, poor; 2, fair; 3, satisfactory; and 4, highly satisfactory); the number of patients with postoperative nausea and vomiting, the toxicity of local anaesthetic;s haematoma at the puncture site and incidence of postoperative epidural volume extension.

### Statistical analysis

The sample size was calculated based on previous trial findings, and the dose of sufentanil was approximately 26.4 ± 4.2 μg (mean ± standard deviation [SD])in the conventional LSPB. We aimed to investigate whether the effectiveness of the anterior approach LSPB was noninferior to that of the conventional LSPB. For the anterior approach LSPB, the standard deviation sufentanil dose was 3.7 μg,and the cut-off value of inferiority (δ) was 2.1 μg. The sample size, calculated by PASS 11.0 software (NCSS, LLC, Kaysville, USA),was 57 individuals per group (with α = 0.025, power = 0.8). Considering the loss-to-follow-up rate of approximately 10%, we enrolled 126 patients.

IBM SPSS Statistics 24.0(Version24; IBM, Armonk, New York) software was used for statistical analysis. Continuous variables were expressed as the mean and variance and analysed using Student’s *t*-test if the data were normally distributed. In cases of nonnormal distribution, the results were expressed as median and range and analyzed with the *Mann–Whitney U* test. The categorical variables were expressed as percentages or numbers and analysed by Pearson’s chi-square tests or Fisher’s exact test. The significance level for all statistical tests was set at *P* < 0.05.

## Results

**Fig. 2:**
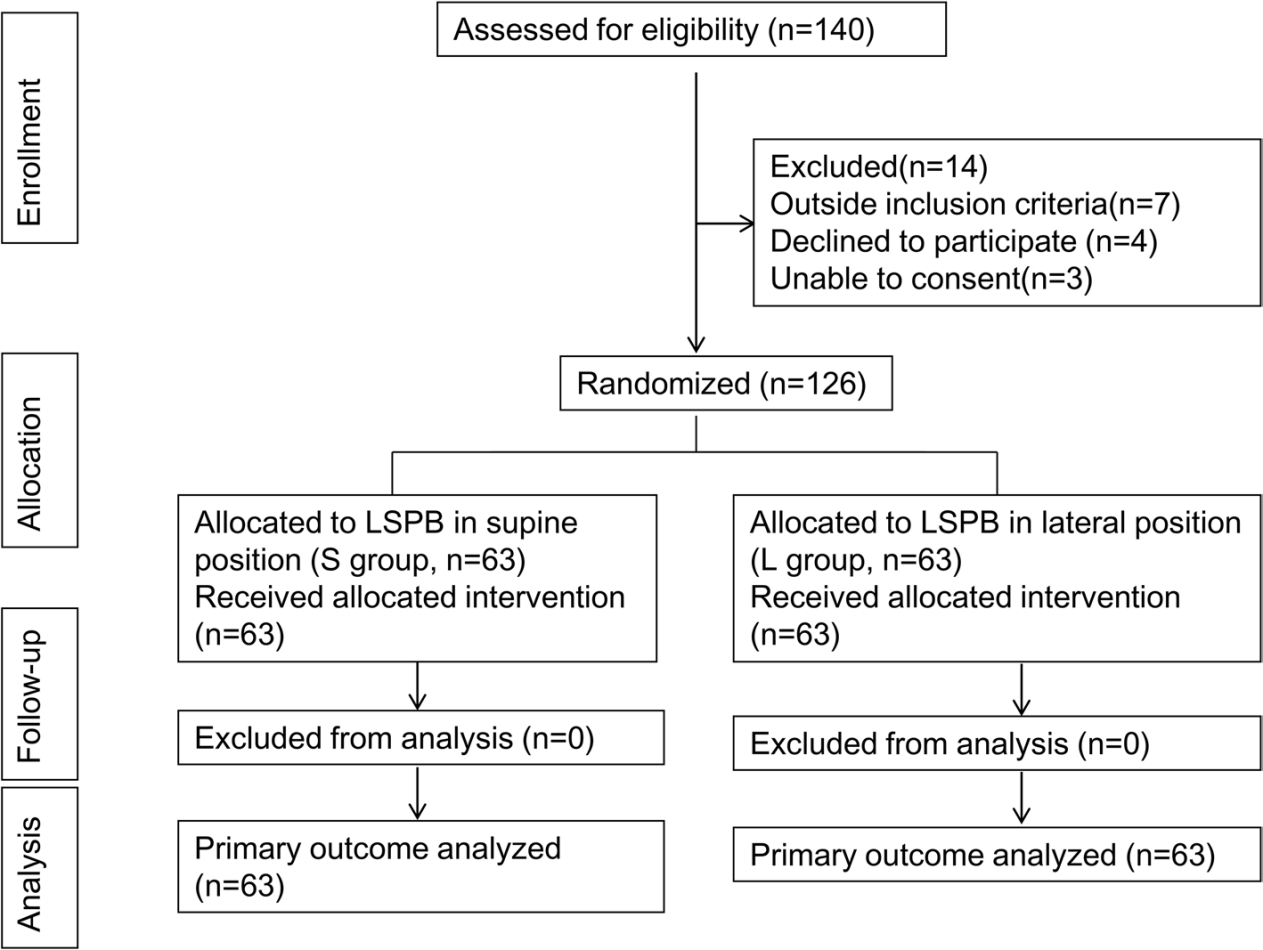
CONSORT diagram of patient flow through the study

A total of 140 patients were screened (Fig. 2), of whom 14 were excluded. A total of 7 out of these 14 patients did not meet the inclusion criteria, 4 declined to participate and 3 were unable to consent. A total of 126 patients were included and randomly assigned to undergo either LSPB in the supine position (*n* = 63) or LSPB in the lateral position (*n* = 63). Eventually, 126 patients completed the study and were analysed as per-protocol (63 in group S, 63 in group L).

### Comparison of the general characteristics of patients between the two groups

A total of 126 patients with the following fractures were included: femoral neck fracture in 26 patients, femoral intertrochanteric fracture in 18 patients, femoral shaft fracture in 20 patients, tibial plateau fracture in 17 patients, tibial and fibula shaft fracture in 30 patients, and malleolar fracture in 15 patients. The age, BMI, ASA grade and sex were not significantly different between the two groups (*P* > 0.05) (Table 1).

**Table 1:** Comparison of general characteristics of the patients between the two groups (mean ± standard)

Parameter	S group (*n* = 63)	L group (n = 63)	*P* value
Age (years)^a^	56.6 ± 15.4	55.8 ± 14.6	0.758
BMI (kg/m^2^)^a^	22.8 ± 1.8	23.0 ± 2.0	0.564
ASA grade I/II (n)^b^	27/36	25/38	0.717
Sex (M/F)^b^	38/25	34/29	0.471

*Abbreviations*: *BMI* Body Mass Index, *ASA* American Society of Anesthesiologists

^a^ Student’s t-test

^b^ χ2 tests or Fisher’s exact tests

### Comparison of the parameters of LSPB and intraoperative sufentanil dose between the two groups

The maximum pain score at position placing, time of position placing, time for lumbar plexus block and the number of puncture attempts for lumbar plexus were significantly lower in the S group than in the L group (*P*_1_ < 0.01, *P*_2_ < 0.01, *P*_3_ < 0.01, *P*_5_ < 0.01). However, the time for sacral plexus block was significantly longer in the S group than in the L group (*P*_4_ = 0.029). The number of puncture attempts for the sacral plexus and the dose of sufentanil used in surgery did not significantly differ between the two groups (*P*_6_ = 0.802, *P*_7_ = 0.142) (Table 2).

**Table 2:** Comparison of parameters of LSPB and intraoperative sufentanil dose between the two groups (mean ± standard, median (25–75 IQ))

Parameter		S group (*n* = 63)	L group (*n* = 63)	*P* value
VAS score at position placing^b^		2.0(1.0,2.0)	4.0(3.0,4.0)	*P*1 < 0.01^*^
Time of position placing (S)^a^		48.8 ± 8.9	113.8 ± 8.0	*P2* < 0.01^*^
Time for nerve block (S)^a^	Lumbar plexus	236.9 ± 12.0	318.7 ± 13.1	*P*3 < 0.01^*^
	Sacral plexus	304.4 ± 11.1	300.3 ± 9.9	*P*4 = 0.029^*^
Time of puncture attempts (time)^b^	Lumbar plexus	1.0(1.0,1.0)	2.0(1.0,3.0)	*P*5 < 0.01^*^
	Sacral plexus	2.0(2.0,3.0)	2.0(2.0,3.0)	*P*6 = 0.802
Dose of sufentanil (μg)^a^		25.1 ± 3.1	26.0 ± 3.0	*P*7 = 0.142

*Abbreviations*: *VAS* Visual analogue scale

^a^ Student’s t-test

^b^ Mann–Whitney U test

^*^ There were significant differences between the two groups (*P* < 0.05)

### Comparison of haemodynamic indicators at different time points between the two groups

The heart rate and MAP at T0, T1, T2, T3 and T4 did not significantly differ between the S group and the L group (*P*_1_ = 0.413, *P*_2_ = 0.656, *P*_3_ = 0.117, *P*_4_ = 0.880, *P*_5_ = 0.642, *P*_6_ = 0.146, *P*_7_ = 0.446, *P*_8_ = 0.688, *P*_9_ = 0.430, *P*_10_ = 0.237) (Table 3).

**Table 3:** Comparison of haemodynamic indicators at different time points between the two groups (mean ± standard)

Parameter		S group (n = 63)	L group (n = 63)	*P*value
Heart rate (beats/min)^a^	T0	75.6 ± 7.5	76.7 ± 7.0	*P*1 = 0.413
	T1	63.6 ± 4.8	64.0 ± 4.7	*P*2 = 0.656
	T2	64.3 ± 4.0	65.7 ± 5.7	*P*3 = 0.117
	T3	68.2 ± 5.0	68.3 ± 5.5	*P*4 = 0.880
	T4	77.7 ± 6.2	78.2 ± 6.8	*P*5 = 0.642
Mean arterial pressure (mmHg)^a^	T0	94.6 ± 9.7	92.1 ± 9.3	*P*6 = 0.146
	T1	86.7 ± 9.8	85.3 ± 10.1	*P*7 = 0.446
	T2	85.1 ± 7.8	85.7 ± 8.2	*P*8 = 0.688
	T3	90.3 ± 9.2	89.0 ± 8.7	*P*9 = 0.430
	T4	95.1 ± 9.9	93.0 ± 9.1	*P*10 = 0.237

^a^ Student’s t-test

### Comparison of the postoperative VAS score and degree of satisfaction with analgesia at different time points between the two groups

The VAS score and degree of satisfaction with analgesia at different time points following the operation were not significantly different between the two groups (*P*_1_ = 0.609, *P*_2_ = 0.361, *P*_3_ = 0.189, *P*_4_ = 0.683) (Table 4).

**Table 4:** Comparison of postoperative VAS score and satisfactory degree to analgesia at different time points between the two groups (median (25–75 IQ))

Parameter		S group (*n* = 63)	L group (*n* = 63)	*P* value
Postoperative VAS score^c^	1 h after operation	1.0(1.0,2.0)	1.0(1.0,2.0)	*P*1 = 0.609
	12 h after operation	3.0(3.0,4.0)	3.0(3.0,4.0)	*P*2 = 0.361
	24 h after operation	5.0(5.0,6.0)	5.0(5.0,6.0)	*P*3 = 0.189
Postoperative satisfactory degree to analgesia^a^		4.0(3.0,4.0)	4.0(3.0,4.0)	*P*4 = 0.683

*Abbreviations*: *VAS* Visual analogue scale

^a^Mann–Whitney U test

### Comparison of nerve block results and adverse events between the two groups

No adverse events, including the toxicity of local anaesthetics, haematoma at puncture sites and postoperative epidural volume extension, were found. Eight patients (12.7%) in the S group and 6 (9.5%) patients in the L group suffered from nausea and vomiting; however, the difference between the two groups was not statistically significant (all *P* > 0.05).

## Discussion

Two main findings can be derived from our prospective randomized controlled trial. First, our results showed that sufentanil consumption was not reduced in the supine LSPB compared with the lateral LSPB,but there was a significant decrease inthe maximum pain score at position placement and time of position placement. Second, supra-inguinal fascia iliaca (SIFI) block more effectively relieved pain, took less time, reduced puncture attempts and decreased the risks of haematoma and nerve injury.

Anaesthesia methods for internal fixation in patients with lower limb fracture included simple general anaesthesia, intraspinal anaesthesia and general anaesthesia combined with a nerve block. Modern anaesthesia should not only ensure successful surgical processes but also take into account the comfort of patients. With the development of visualization technology and the application of nerve stimulators, nerve block [15] has become increasingly and widely used in clinical practice due to specific advantages, including high safety, low invasiveness and few side effects. General anaesthesia combined with nerve block has been widely used in orthopaedic surgeries due to its well-recognized advantages A study by Yuan H demonstrated that compared to general anaesthesia with intubation and combined spinal-epidural anaesthesia, general anaesthesia with LMA and nerve block had better postoperative analgesic effects and fewer disturbances on intraoperative haemodynamics and postoperative cognition for elderly patients undergoing intertrochanteric fracture surgeries [16].

In our study,there was no statistically significant difference in sufentanil consumption between the two groups. Accumulating published data [17–19] have been dedicated to exploring more effective multimodal analgesia with opioid-sparing. Daniela’s study [20] showed that LPB and SIFI block in terms of breakthrough morphine requirement and pain control are the same, and SIFI block resulted in a longer block and was associated with shorter time to readiness for discharge as well as decreased hospital stay. When an SIFI block is combined with a sacral plexus block, almost the whole area of the buttock, perineum and limb can be blocked, which can satisfy the anaesthetic requirements for lower limb surgery. The branches of the lumbar plexus are the iliohypogastric nerve, ilioinguinal nerve, femoral nerve, lateral femoral cutaneous nerve and obturator nerve. The main branches of the sacral plexus are the superior gluteal nerve, inferior gluteal nerve, pudendal nerve, sciatic nerve and posterior femoral cutaneous nerve. LSPB blocks the above nerves, and can achieve the blocking effect of unilateral spinal anaesthesia. Furthermore, LSPB can provide adequate postoperative analgesia, enabling patients to get out of bed early for routine activities and exercises to strengthen joint function. Badiola et al. [21] suggested that the analgesic effect of the SIFI block was similar to that of the lumbar plexus block. As we found in our study, postoperative VAS scores and postoperative satisfactory degree to analgesia did not differ between the two groups.

Conventional LSPB is performed in the lateral position, where patients are required to engage in position changing and is therefore not convenient for patients treated with an external fixator. The classic anterior approach for blocking the lumbar plexus [22] could easily lead to abdominal visceral injuries due to the deep position, which could in turn induce complications, such as epidural diffusion of local anaesthetics and vertebral canal anaesthesia [23]. Fascia iliaca compartment block (FICB) has been defined as an anterior approach of the lumbar plexus block method, which is easy to perform and exhibits high safety and optimal analgesic effects. Wennberg [24] et al. reported that FICB effectively provided high-quality pain relief after THA. It has become increasingly accepted and is widely used in lower limb surgeries in orthopaedics departments [25]. Previous studies have reported that the failure rate of FICB, which aimed to block the lateral femoral cutaneous nerve, was approximately 10–37% due to the individualized variation [26], distribution and branching of nerves inferior to the inguinal ligament. Despite this evidence, the selection of the area above the inguinal ligament and below the pelvic iliac fascia is considered a reliable approach. Therefore, an applicable method modified from FICB, termed SIFI block, was developed to replace the conventional fascia iliaca block and femoral nerve block [27]. Zheng et al. [9] used the SIFI compartment block to demonstrate that the diffusion of 30 ml local anaesthetics could fully block the femoral nerve and lateral femoral cutaneous nerve, and provide a 56% block of the obturator nerve. Our study indicated that the maximal pain score and time of position placement were significantly lower in the S group than in the L group, which suggested that the supine position had less pain and less time consumption. We speculated that this could be due to the following reasons: 1) the stimulation during position placement could increase the pain and require cooperation among multiple operators; and 2) the procedures of position placement in patients using an external fixator were more complex and more time-consuming and thus increased the pain stimulation. A lower pain score indicates better control of acute pain and can reduce the risk of developing chronic pain, shorten recovery, and better quality of life [28].

The SIFI compartment block involves several features, including superficial location and rapid and clear ultrasound imaging compared with the conventional posterior approach lumbar plexus block [20]. The superficial layer of the fascia iliaca compartment was covered by fascia lata and fascia iliaca and the deep layer was the iliopsoas muscle, through which the femoral nerve and lateral femoral cutaneous nerve were allowed to travel. The high-frequency linear array probe has a high resolution for superficial tissues and the images are very clear. Therefore, ultrasound-guided procedures could easily aid the injection of local anaesthetics to target specific sites, resulting in fewer puncture attempts [29, 30]. Due to the deep position and complex anatomical structures of the lumbar plexus, the block was relatively difficult [31]. As the fascial plane block target is a fascial plane rather than a specific nerve (nerve root), this approach decreases the risk of nerve injury [32]. The injection site of the needle tip is more superficial, which reduces the risk of unrecognized blood vessel bleeding [33]. Consistent with our findings, SIFI block had better pain relief, less time consumption, fewer puncture attempts and a lower risk of haematoma and nerve injury than patients in group L.

The anatomical position of the sacral plexus was deeper in the supine position and the puncture was more difficult. Generally, such procedures need to be performed by experienced and skilled anaesthetists to identify bone landmarks, such as the anterior superior spine. Increased pressure is generally required during the procedures to reduce the thickness of subcutaneous adipose tissues, which makes the imaging of the deep nerves more sufficient. In addition, the bed was adjusted to uplift the affected body area of the patient by 15°, which increased the operation field, facilitated the procedures and saved time.

In a case report, ultrasound-guided SIFI combined with a sacral plexus block was found to be suitable for anaesthesia for patients with severe circulatory compromise and avoided all haemodynamic fluctuations [27]. In our study,intubation via laryngeal mask was performed 30 min following nerve block for patients in both groups, which shortened the onset time of the nerve block effects. The onset of the nerve block effect could provide sufficient analgesic effects, thus meeting the surgery requirements and aiding patients in tolerating surgical stimulations, while the effects on haemodynamics were not substantial.

The present study contains certain limitations. The data were derived from two hospitals, which may limit the generalizability of our results. The follow-up investigator may have been biased. The blockers were two anaesthetists and may have performed operational errors. Considering the limitations of the two hospitals, we try to ensure consistency in other aspects. The same ultrasound (FUJIFILM SonoSite, Bothell, WA98021 USA) was used in both hospitals, and the choice of USG probe (high frequency/low frequency) for each step was also consistent.

## Conclusions

Ultrasound combined with nerve stimulator-guided LSPB in the supine position is a more comfortable and better accepted anaesthetic regimen for patients undergoing lower limb fracture surgery. LSPB in the supine position is simple to apply and has definite anaesthetic effects. Additionally, it has a high level of postoperative analgesia, maintains haemodynamic stability and is accompanied by few side effects [34]. Therefore, LSPB in the supine position is considered applicable in clinical practice and worth wide application.

## Supplementary Information

**Additional file 1.**

## Data Availability

The datasets used and/or analysed during the current study are available from the corresponding author and editorial office on reasonable request.
