# Supplementary material for: Applicability and effectiveness of ultrasound combined with nerve stimulator-guided lumbosacral plexus block in the supine versus lateral position during surgeries for lower limb fracture-a prospective randomized controlled trial
Source: BMC Anesthesiol. 2022 Jun 3;22:174. doi: 10.1186/s12871-022-01710-9 (PMC9164414; doi:10.1186/s12871-022-01710-9)
Supplement: Supplementary file 1 — Additional file 1. [file 12871_2022_1710_MOESM1_ESM.docx]

***Research protocol***

***Project summary***

**Rationale**:To compare the applicability and effectiveness of ultrasound combined nerve stimulator-guided lumbosacral plexus block (LSPB) in supine versus lateral position during the lower limb fracture surgery.

**Objectives**:A total of 126 patients with the following fractures were recruited at the department of Anesthesiology in two affiliated hospitals of Anhui Medical University (Chaohu Hospital, Chaohu; and the Second Hospital, Hefei). The patients were individually randomized by the hospital. The patients who underwent elective internal fixation for unilateral lower limb fracture (ascertained using radiological reports from the medical imaging centre, at Chaohu Hospital and the Second Hospital) between March 2021 and June 2021, were included

**Methods**:Patients who underwent elective internal fixation for lower limb fracture were divided into the S group and the L group by the random number table method and received LSPB guided by ultrasound combined nerve stimulator in supine and lateral positions, respectively. The primary outcome examined was the dose of sufentanil used in surgery and the secondary outcomes were the maximum VAS pain score at position placing for LSPB, the time of position placing, the time for nerve block and the number of puncture attempts.

**Expected outcomes**: Firstly, LSPB in the supine position could reduce the maximum pain score at position placing and time of position placing. Secondly, supra-inguinal fascia iliaca (SIFI) block shortened the time periods required for nerve block and puncture and reduced the possibility of tissue injury and nerve damage compared with those noted in the traditional lumbar nerve block.

***General information***

**Protocol title**: Applicability and effectiveness of ultrasound combined nerve stimulator-guided lumbosacral plexus block in supine versus lateral position during surgeries for lower limb fracture-A prospective randomized controlled trial

The trial was registered prior to patient enrollment at the Chinese Clinical Trail Registry (2021/03/11 ChiCTR2100044117).The present study was approved by the Ethics Committee of the Chaohu Hospital Affiliated to Anhui Medical University (2020/12/01 202001-KYXM-01).

**Funding**: None

**Project leader**: Xiaoqiong Xia, Xianwen Hu

**Members of the project team**: Yuting Xu , Jie Song, Yawen Li, Yongbo Yu, Liang Wang, Zhiguo Tao.

**Research site**:Department of Anesthesiology, Chaohu Hospital Affiliated to Anhui Medical University, Chaohu, Anhui, China

Department of Anesthesiology，the Second Hospital Affiliated to Anhui Medical University, Hefei, Anhui, China

Xiaoqiong Xia Email: xxq2366833@sina.comTel: +86-13966386669

Xianwen Hu Email: huxianwen001@163.comTel: +86-15155159719

Fax: 056582324034

YX: Contributed substantially to study conception and design, execution of study, data acquisition, analysis, data interpretation first drafting. JS: Contributed substantially execution of study, data acquisition, interpretation of results. YL: Contributed substantially execution of study, data acquisition. YY: Contributed substantially execution of study, data acquisition. XX: Contributed substantially to study conception and design, and critical revision of the manuscript. XH: Contributed substantially to study conception and design, and critical revision of the manuscript. LW: Contributed substantially execution of study, interpretation of results. ZT: Contributed substantially to interpretation of data and critical revision of the manuscript. All authors read and approved the final manuscript.

***Rationale & background information***

Lower limb fractures account for approximately one third of all fractures and may result in substantial mortality and morbidity [1] The most common anatomic position of lower limb fracture is the ankle joint, which accounts for 22.6% of all lower limb fractures, followed by tibia/fibula (17.3%), hip joint (16.7%) and tarsal/metatarsal bone (16.7%). Fractures of the hip, femur and other parts account for approximately 25% of all fractures [2].

Rapid urbanization and accelerated aging of the population in China have led to a rapid increase in the number of patients with fractures caused by traffic injuries, architecture injuries and senile osteoporosis. The increase in residual injuries and disability increases the potential life loss and thus has become an important public health concern [3]. Lower limb fractures include various types and are usually caused by large wounds. Therefore they generally require correction by surgery.

Over the recent years, lumbosacral plexus block (LSPB) has been widely applied in orthopedics departments due to the advantages, including reduction in the application of opiates, decreasing the occurrence of acute pain, promoting early activation and shortening the time of hospital stay [4]. De Visme et al. has proved that LSPB exhibit an advantage of limited sympathectomy and bladder paralysis [5]. Ultrasound-guided nerve blocks with nerve stimulators increase the success rate and reduce risks, such as nerve injuries, undesirable spread-hematoma and renal puncture [6] The conventional LSPB is mainly performed in the lateral position, while only very few studies have reported LSPB in the supine position. Therefore, the present study aimed to compare the applicability, anesthetic effects and postoperative analgesic effects of supine versus lateral LSPB under the guidance of ultrasound combined nerve stimulator and provide evidence for the application of LSPB in a supine position for anesthesia in clinical practice.

***References***

1.Holloway KL, Yousif D, Bucki-Smith G,et al. Lower limb fracture presentations at a regional hospital. Arch Osteoporos 2017; 12(1):75.

2.Kaye JA, Jick H. Epidemiology of lower limb fractures in general practice in the United Kingdom. Inj Prev 2004; 10(6):368-74.

3.Singh A, Lim ASM, Lau BPH, O'Neill G. Epidemiology of pelvic and acetabular fractures in a tertiary hospital in Singapore. Singapore Med J 2021.

4.Yang R, Liu RH, Xu JN,et al. Effects of Different Local Analgesic Techniques on Postoperative Quality of Life and Pain in Patients Undergoing Total Hip Arthroplasty Under General Anesthesia: A Randomized Controlled Trial. J Pain Res 2021; 14:527-36.

5.de Visme V, Picart F, Le Jouan R,et al. Combined lumbar and sacral plexus block compared with plain bupivacaine spinal anesthesia for hip fractures in the elderly. Reg Anesth Pain Med 2000; 25(2):158-62.

6.Kirchmair L, Entner T, Kapral S, Mitterschiffthaler G. Ultrasound guidance for the psoas compartment block: an imaging study. Anesth Analg 2002; 94(3):706-10; table of contents.

***Study goals and objectives***

The primary outcome was (1) the dose of sufentanil used in surgery. The secondary outcomes were (2) the maximum VAS pain score at position placing for LSPB (1-10 points: 0 point indicated no pain and 10 points indicated drastic pain); the time of position placing, the time for nerve block (from skin anesthesia, ultrasound imaging, to the completion of local anesthetics injection) and a number of puncture attempts (each withdrawal of the needle to adjust the direction was considered as one attempt of puncture); (3) the hemodynamic indicators, including heart rate and arterial pressure and the observation time including the time of entering the operating room (T0), completion of a nerve block (T1), skin incision (T2), skin suturing (T3) and 30 min following completion of the surgery (T4); (4) the postoperative VAS score, with the higher scores indicating more severe pain. The VAS score was evaluated at 1, 12, and 24 h following surgery; (5) the postoperative satisfactory degree to analgesia, where the scores ranged from 1 to 4 points (1, poor; 2, fair; 3, satisfactory; and 4, highly satisfactory); (6) the number of patients with postoperative nausea and vomiting, the toxicity of local anesthetics, hematoma at the puncture site and incidence of postoperative epidural volume extension.

***Study design***

This trial was a randomized controlled single blinded clinical trial. The patients were blind to the group allocations. Group assignments were concealed in opaque envelopes until consent had been obtained.

**Inclusion and exclusion criteria**

**The inclusion criteria used were the following**: 1) Patients diagnosed with unilateral femoral neck fracture or lower fractures by X-ray or CT examination; 2) patients who consented to participate in the study and signed the relevant informed consent form (a study physician approached the patient in the preoperative area and informed consent was obtained preoperatively if he or she elected to proceed with the study); 3) patients with available complete clinical data and 4) patients with the capability of communication, expression and comprehension.

**The exclusion criteria were the following**: 1) Patients with mental disorders or psychonosema; 2) patients accompanied with renal, liver or cardiac insufficiency; 3) patients with coagulation disorders; 4) patients who refused to participate or withdrew due to personal reasons.

***Methodology***

All the patients received vital sign monitoring and the venous channel was established. intravenous pumping of Dexmedetomidine was performed at the rate of 300 μg/h for 10 min to induce the full sedation of the patients. Subsequently, LSPB was performed under the guidance of ultrasound combined nerve stimulator, using nerve stimulator with 5-10 MHz high-frequency linear array probe and 2-5 MHz low-frequency convex array probe in supine and lateral positions, respectively. Following completion of the nerve block in both groups, venous anesthesia was performed. In brief, 0.2 μg/kg sufentanil and 1.5-2 mg/kg propofol were used for anesthesia induction. Intubation was performed via laryngeal mask based on a BIS value of <60 to allow autonomous or controlled respiration. For anesthesia maintenance, venous pumping of propofol [4-12 mg/(kg·h)] was performed. According to the hemodynamics, 5 μg sufentanil was added if the heart rate or arterial pressure was increased by 15% in the surgery. Following surgery, an analgesia pump was used for analgesia.

***Safety considerations***

All patients gave their written informed consent before participation.

Local anesthetic poisoning. Treatment 1. Stop the injection of local anesthetics.2. Protect the airway, give pure oxygen, and conduct endotracheal intubation if necessary to control the airway to avoid hypoxemia and hypercapnia.3. To inhibit convulsions, imidazolepam or propofol can be selected, and the small dose is gradually increased to effectively control convulsions.4. Start using 20% fat milk (intralipid) as soon as possible 5. Use of volume recovery and positive muscle strength drugs and vasoactive drugs to maintain hemodynamic stability in patients with local anesthesia poisoning may need longer circulatory support to drug metabolism discharge.6. Treat arrhythmias and treat amiodarone with ventricular armias.Pharmacotherapy with ineffective ventricular tachycardia was considered for electrocardioversion.7. Start the cpr process immediately in case of cardiac arrest.During general anesthesia, if the patient had a heart rate 50 times, atropine, blood pressure was 90 / 60mmhg and ephedrine was used.

***Data management and statistical analysis***

IBM SPSS Statistics 24.0(Version24; IBM, Armonk, New York) software was used for statistical analysis. Continuous variables were expressed as mean and variance and analyzed using an independent samples t-test if the data were normally distributed. In case of non-normal distribution, the results were expressed as median and range and analyzed with the Mann–Whitney U test. The categorical variables were expressed as percentages or numbers and analyzed by the Pearson’s Chi-square tests or the Fisher’s exact test. The significance level for all statistical tests was set at P< 0.05.

The present study was a randomized control trial with a non-inferiority design, which aimed to investigate whether the effectiveness of the anterior approach LSPB was not inferior to the conventional LSPB. According to the previous trial findings, the dose of sufentanil was approximately 26.4±4.2 μg in the conventional LSPB. For the anterior approach LSPB, the standard sufentanil dose was 3.7 μg for the anterior approach LSPB. The cut-off value of inferiority (δ) was 2.1 μg, whereas the α value was 0.025 (one-sided) and the power (1-β) was 0.8. The numbers of subjects in the two groups were equal. PASS 11 software was used to estimate the sample size in the study and control groups, generating N1=N2=57. A drop-off ratio of 10% was considered and the final sample size was 126 (63 per group).

***Duration of the project***

January 2021 to February 2021: study conception and design.

March 2021 to June 2021: execution of study ,data acquisition.

July 2021 to October 2021: analysis, data interpretation first drafting, critical revision of the manuscript.

November 2021 to December 2021: submit manuscript.

***Informed consent forms***

Informed consent form was developed.See documentation for details.

***Budget***

All drug,ultrasound, nerve stimulator in this experiment were provided by the anesthesiology department .With no additional budget.

***Other support for the project***

None
